# Early and late outcomes of a series of 255 patients with Crohn’s disease who underwent resection: 10 years of experience at a single referral center

**DOI:** 10.1007/s13304-022-01322-5

**Published:** 2022-07-16

**Authors:** Franco Sacchetti, Paola Caprino, Angelo Eugenio Potenza, Dario Pastena, Stefano Presacco, Luigi Sofo

**Affiliations:** 1 Abdominal Surgery Unit, Fondazione Policlinico Universitario “A. Gemelli”-IRCCS, Largo Agostino Gemelli, 8, 00168 Rome, Italy; 2 Università Cattolica del Sacro Cuore di Roma, Rome, Italy

**Keywords:** Crohn’s disease, IBD surgery, Malnutrition, Crohn’s disease surgery

## Abstract

Patients with Crohn's disease experience an increased risk of postoperative complications and disease recurrence. The aim of this study was to investigate the role of the risk factors in determining these outcomes and whether preoperative removal of some of these risk factors would optimize the results. We conducted a retrospective study analyzing a consecutive series of 255 patients who underwent surgical resection for Crohn's disease between 2010 and 2020. We considered short- and long-term endpoints, such as postoperative complications categorized according to the Clavien–Dindo classification and the appearance of surgical and endoscopic postoperative recurrence. Univariable and multivariable analyses showed that multiple and extensive localizations increased the incidence of postoperative complications (OR = 2.19; 95% CI 1.05–4.5; *p* = 0.035 and OR = 1.015; 95% CI 1.003–1.028; *p* = 0.017 for each cm of resected segment, respectively). Regarding theoretically modifiable factors, preoperative hypoalbuminemia (for each g/L reduction) increased the risk of complications with an OR = 1.1; 95% CI 1.02–1.12; *p* = 0.003. Preoperative steroid therapy exerted a similar effect, with an OR = 2.6; 95% CI 1.1–5.9; *p* = 0.018. Modifying these last two risk factors by improving the nutritional status or discontinuing steroid therapy significantly reduced complications.

Microscopic positivity of the resection margins was a risk factor for surgical recurrence (OR = 8.7; 95% CI 1.9–40; *p* = 0.05). Based on the results of the present study, surgeons must examine modifiable risk factors, and careful preoperative tailored management may reduce postoperative complications and disease recurrence.

## Introduction

Crohn’s disease (CD) is a chronic inflammatory disease potentially involving the whole gastrointestinal tract, characterized by relapsing episodes of active inflammation alternating with periods of disease remission [1].

In patients with inflammatory manifestations localized to the mucosa, pharmacological treatment can control the disease; however, CD, in its natural history, can evolve towards stenosing or fistulising patterns with a subsequent need for surgical treatment (up to 70–80% of CD patients 20 years after diagnosis) [1].

This finding becomes even more relevant because almost 65% of operated patients require at least a second surgical intervention due to recurrence [2].

In addition to being burdened by a high relapse rate, CD surgery has a rate of postoperative complications that, depending on the severity, can even reach 40% [3].

The presence of specific risk factors, some of which have already been reported in the literature, significantly influence this rate.

Although some risk factors associated with increased postoperative complications are not modifiable (e.g., clinical presentation, disease pattern, and intra-abdominal abscesses), others can be modified and optimized preoperatively, such as malnutrition, which is frequent in patients with CD, or preoperative pharmacological therapy.

The aim of the study was to retrospectively investigate which factors are associated with an increased incidence of postoperative complications and disease recurrence and highlight whether tailored preoperative strategies were able to improve the outcomes.

## Patients and methods

Consecutive patients with CD who underwent resection surgery between January 2010 and August 2020 were included in this retrospective study.

Patients with ileal, ileocolic, colic or rectal localizations who underwent first or second surgery for recurrence of disease were included.

Patients who performed only non-resective surgery (e.g. ileostomy closure, stricture plasties alone or intestinal by-pass) or interventions for perianal CD were excluded.

Data were collected from medical, outpatient and operating records. Each patient was included in an outpatient/telephone follow-up.

For each patient, the following characteristics were recorded: age, sex, preoperative nutritional status, type and site of the disease, preoperative drug therapy, type and characteristics of surgery performed, duration and type of preoperative nutrition, timing of preoperative drug suspension and other factors, as presented in Table 1.

**Table 1:** Patient characteristics

	n	Frequency (%)
Sex ratio (F:M)	121: 134	47.5: 52.5
Smoking at time of surgery	69	27.1
Nutritional status		
BMI<18.5	65	25.5
Preoperative hypoalbuminemia (< 35 g/L)	111	43.5
Severe preoperative hypoalbuminemia (< 25 g/L)	10	3.9
Malnourished patients	101	39.6
Medical therapy at surgery		
Steroids	28	11
Anti-TNF α	24	9.4
Behaviour		
B2 (stricturing)	122	47.8
B3 (penetrating)	133	52.1
Preoperative intra-abdominal abscess	56	22
Location		
Small bowel	26	10.2
Colon	25	9.8
Rectum	8	3.1
Ileo-cecal	159	62.3
Multiple locations	37	14.5
Concomitant perianal disease	70	27.4
Preoperative fever	17	6.7
Recurrent disease (previous bowel resections)	91	35.7
Urgent surgery	31	12.2
Surgical procedure		
Ileo-cecal resection	160	62.7
Small bowel resection	11	4.3
Right hemicolectomy	6	2.4
Left hemicolectomy	10	3.9
Total colectomy	22	8.6
Combined resections	42	16.5
Proctectomy	4	1.6
Laparotomy	80	31.4
Laparoscopy	116	45.5
Lps/Lpt conversion	59	23.1
Hand-sewn anastomosis	168	65.9
Stapled anastomosis	44	17.2
Intracorporeal anastomosis	27	10.6
Histological margin positivity	101	39.6

The primary endpoint was the occurrence of postoperative complications of grade > 1 s. the Clavien–Dindo classification (C-D complications) within 30 days of surgery (Table 2).

**Table 2:** Clavien‒Dindo classification

Grade	
1	Any deviation from the normal postoperative course without the need for pharmacological treatment or surgical, endoscopic and radiological interventions. Acceptable therapeutic regimens are: drugs as antiemetics, antipyretics, analgesics, diuretics and electrolytes and physiotherapy. This grade also includes wound infections opened at the bedside.
2	Requiring pharmacological treatment with drugs other than such allowed for grade I complications. Blood transfusions, antibiotics and total parenteral nutrition are also included.
3	Requiring surgical, endoscopic or radiological intervention
3a	Intervention under regional/local anesthesia
3b	Intervention under general anesthesia
4	Life-threatening complication requiring intensive care/intensive care unit management
4a	Single organ dysfunction
4b	Multi-organ dysfunction
5	Patient demise

The secondary endpoints were severe postoperative complications (C-D complications > 2), postoperative surgical recurrence (defined as the need of further surgery for disease recurrence), and other complications (as reported in Table 3).

**Table 3:** Postoperative complications considered during the study

	*n*	Percentage (%)
Primary endpoint		
Postoperative complications (Clavien-Dindo >1)	91	35.7
Secondary endpoints		
Postoperative complications (Clavien-Dindo >2)	23	9
Anastomotic leak	12	5.6
Reoperation	20	7.8
Intestinal obstruction	12	4.7
Postoperative fever	74	29
Preoperative intra-abdominal abscess	29	11.4
Postoperative sepsis	14	5.5
Surgical recurrence	16	6.3
Endoscopic recurrence	59	23.1

### Statistical analysis

The statistical analysis was conducted using SPSS software (v. 24.0, Inc., Chicago, IL).

Categorical data are presented as absolute and percentages and were compared using the *χ*^2^ test.

Logistic regression analyses were used to investigate the correlation between risk factors and postoperative complications; the results are reported as odds ratios with 95% confidence intervals.

The level of statistical significance was set to *p* = 0.05.

## Results

Two hundred fifty-five patients (47.5% females) were included in the study.

The mean duration of follow-up was 40.3 months (minimum period of 14 months).

Approximately 27% of patients were smokers at the time of surgery.

One hundred thirty-three patients (52.2%) had a fistulising pattern, and 56 of them had preoperatively documented intra-abdominal abscesses.

The most frequent localization of disease was ileocecal (62.4%), and over ¼ of the total patients had concomitant perianal disease.

A total of 35.7% of patients had already undergone previous surgery for CD.

A total of 39.6% of patients had a clinical presentation of malnutrition defined according to the ESPEN 2017 criteria: weight loss > 10% within 6 months, BMI < 18.5 kg/m^2^ or serum albumin level < 30 g/L (with no evidence of hepatic or renal dysfunction) [4].

Twenty-eight patients (11%) were taking steroid therapy within 7 days before surgery.

### Postoperative complications

Related to the primary endpoint, 91 patients (35.7%) experienced a grade C-D > 1 postoperative complication (Table 3).

#### Malnutrition

65 of 255 patients had a BMI < 18.5 at the time of surgery. This group of patients showed a higher C-D > 1 postoperative complication rate than controls (46.2% vs. 32.6%; *p* = 0.04) as shown in Table 4.

**Table 4:** Postoperative C-D complications > 1

Frequency (%)	*p*
38.1–33.1	0.40
30.4–37	0.54
46.2–32.3	0.04
60–35.9	0.12
42.6–31.2	0.06
57.1–33.5	0.04
33.3–36.8	0.74
37.5–35.2	0.07
58.8–36.4	0.09
36.3–35.4	0.89
41.9–34.8	0.44
30	–
46	–
40	–
30	–
55	–
45	–
38.8–34.3	0.49
31–39.6	0.16
40.7–34.2	0.36
33	0.07
32	
25.9–33.5	0.43
85.7–35.7	< 0.0005

Patients with severe hypoalbuminemia at the time of surgery (< 25 g/L; *n* = 10; 3.9%) showed a significant increase in postoperative intra-abdominal abscesses (50% vs. 10.1%; *p* = 0.003) and postoperative anemia (50% vs. 13.5%; *p* = 0.002).

In relation to the primary outcome, the univariable analysis showed that for each g/L decrease in the albumin level, a 1.10-fold increased risk of developing a C-D complication > 1 was observed (*p* = 0.003 and 95% CI = [1.02; 1.12]).

These data were confirmed in the multivariable analysis with an OR = 1.06. 95% CI [1.006; 1.13] (Table 5).

**Table 5:** Univariable and multivariable regression analysis postoperative C-D complications > 1

	Univariable regression analysis			Multivariable regression analysis	
	OR	*p*	95% CI	OR	95% CI
Preoperative Steroid Therapy	2.6	0.018	1.1; 5.9	–	–
Preoperative serum albumin (to the reduction of each g/L)	1.1	0.003	1.02; 1.12	1.06	1.006; 1.13
Multiple disease location (compared to ileocecal location)	2.19	0.035	1.05; 4.5	–	–
Surgical intervention duration (increasing every minute)	1.005	0.003	1.001; 1.009	–	–
Length of surgical specimen (increasing every cm)	1.015	0.017	1.003; 1.028	–	–
Intraoperative blood transfusion	10.8	0.02	2.5; 50	–	–

Increases in the incidence of anastomotic leaks (11.1% vs. 5.6%), the need for reoperation during the same hospitalization (20% vs. 7.6%) and C-D complications > 2 (20% vs. 8.9%) were also observed, but the differences did not reach statistical significance.

#### Pharmacological therapy

We did not document statistically significant relationships between the intake of biological drugs in the 8 weeks before surgery and an increase in the incidence of postoperative complications of any nature (33.3% vs.36.8%; *p* = 0.74).

The group of patients who took corticosteroids at the time or within 7 days before surgery, on the other hand, showed a higher rate of postoperative complications C-D > 1 (57.1 vs. 33.5; *p* = 0.04).

The univariable analysis showed that steroid drugs represent a risk factor for the onset of postoperative C-D complications > 1 with an OR of 2.6 (*p* = 0.018 and 95% CI = [1.1; 5.9]).

#### Other risk factors

Patients with CD in more than one location (simultaneous presence of CD in at least two of the following regions: tenual, colic, and rectal) had a 2.19 times higher risk of developing postoperative C-D complications > 1 than patients with an ileocecal localization (*p* = 0.035 and 95% CI = [1.05; 4.5]).

A similar result was observed for the length of the specimen; the risk of C-D complications > 1 increased 1.015 times for each centimeter of pathological segment removed during surgery (*p* = 0.017 and 95% CI = [1.003; 1.028]).

Based on the logistic regression models studied, the risk of developing postoperative C-D complications > 1 increases by 1005 times for each minute of duration of surgery (*p* = 0.003 and 95% CI = [1.001; 1.009]).

### Preoperative optimization and postoperative complications

#### Malnutrition

Among the 101 patients with preoperative clinical malnutrition, 59 underwent a preoperative nutritional program (enteral, parenteral or both) for a mean duration of 42.9 days.

The preoperative nutrition group showed a lower incidence of postoperative C-D complications > 2 (8.5% vs. 19%; p = 0.04), a lower incidence of anastomotic leaks (0% vs. 16.7%; *p* = 0.004) and a lower need for reoperation during the same hospitalization (5.1% vs. 19%; *p* = 0.027). Consequently, these patients had a shorter mean length of hospital stay (7.83 vs. 8.94 days).

#### Pharmacological therapy

Of the 57 patients who received steroid therapy at the time of surgical indication, 29 discontinued treatment at least 7 days before surgery. Discontinuation was not possible for the remaining 28 patients for various reasons largely related to urgent clinical conditions, and they underwent surgery while taking steroid drugs.

C-D complications > 1 occurred in the first group at less than half of the value observed in the second group (24.1% vs. 57.1%; *p* = 0.02).

## Recurrence

59 patients (23.1%) experienced postoperative endoscopic recurrence of CD. Of these 16 patients (6.3% of the total) required further surgery for surgical recurrence of disease during the follow-up.

In the multivariate analysis we identified the concomitant presence of perianal disease and the presence of microscopic positivity of the resection margins as risk factors for surgical recurrence of disease (the latter factor also confirmed in the multivariate with OR = 8.7; *p* < 0.05) as reported in Table 6.

**Table 6:** Univariable and multivariable regression analysis of surgical recurrence

	Univariable regression analysis			Multivariable regression analysis	
	OR	*p*	95% CI	OR	*p*
Perianal disease	3.1	0.03	1.12; 8.9	–	–
Microscopic margin (positive)	8.7	0.05	1.9; 40	8.7	0.0005

## Discussion

The heterogeneous clinical presentation, prolonged immunosuppressive therapy and malnutrition of patients with CD exert a substantial effect on the quality of life of patients and potentially influence postoperative outcomes.

Therefore, careful and multidisciplinary management of the disease of each patient is mandatory and should aim to coordinate multiple therapeutic strategies to optimize short- and long-term outcomes.

From a surgical perspective, difficulties arise both preoperatively because of the sequelae of therapies (e.g., steroids), malnutrition and global impairment of the patient's health and intraoperatively because of strict anatomopathological aspects (abscesses and fistulas, tissue fragility, increased bleeding, mesenteric thickening, lymphadenomegaly, and adhesions due to reoperations for recurrence).

A well-conducted surgical procedure may be ineffective if apparently secondary aspects, such as the patient's nutritional conditions, recent intake of specific therapies or fistulising nature of the disease with intra-abdominal abscesses, are neglected.

## Malnutrition

Malnutrition is a frequent condition in patients with CD. It is often difficult to identify because a single parameter is unable to define malnutrition.

In clinical practice, nutritionists usually evaluate laboratory and biometric parameters (hypoalbuminemia, hypocholesterolemia, lymphocytopenia and BMI) and weight loss due to malabsorption or inadequate oral feeding for mechanical or psychological reasons that often result in anorexic habits.

Low serum albumin levels were observed in 25–50% of patients with CD requiring hospitalization.

During inflammation, protein synthesis (including albumin synthesis) in the liver may be compromised to the advantage of the production of proinflammatory cytokines; in addition, a condition of increased protein dispersion has been linked to increased vascular permeability [5].

Accumulating scientific evidence associates low preoperative albumin levels with worse postoperative outcomes.

Among these studies, a recent study conducted on 6082 patients with CD reported a greater number of postoperative complications, with particular reference to septic complications (20% vs. 13%), in patients with preoperative hypoalbuminemia [6].

## Pharmacological therapy

If a prudent attitude has been adopted regarding the preoperative use of biological drugs in the last decade, which is attributed to the increasing incidence of postoperative complications [7], some recent studies have not confirmed significant associations between preoperative biological therapy and any type of postoperative complications [8].

Our experience endorses this scientific evidence because we did not document statistically significant relationships between the intake of biological drugs in the 8 weeks before surgery and an increase in the incidence of postoperative complications of any nature.

In contrast, the association between preoperative steroid therapy and an increased incidence of postoperative complications is well documented in numerous published studies [9], and our results confirmed this evidence, documenting a significant increase in postoperative complications in the group of patients on steroid drug therapy at the time of surgery.

## Modifiable risk factors

In our population, intervening to optimize some preoperatory risk factors, we noticed a significant improvement in postoperative outcomes.

This finding adds further value to the importance of the risk factors mentioned above and opens up the possibility that their preoperative optimization when possible can result in a real and tangible improvement in the postoperative course.

Our experience in this study is undoubtedly conditioned by many limitations, first of all the retrospective nature of the study, but it can nevertheless serve as a starting point and encouragement to pay more and more attention to the general preoperative conditions of each individual patient to intervene through specific preoperative treatments.

Malnourished patients subjected to a cycle of variable duration of preoperative nutrition (parenteral or enteral nutrition) presented a numerically lower rate of serious postoperative complications, fewer re-operations during the same hospitalization and a shorter length of the postoperative hospital stay.

The same trend was observed among patients for whom it was possible to promptly discontinue steroid therapy: they presented fewer postoperative complications C-D > 1, distancing surgery from the harmful effects of steroid therapy.

These preoperative measures are not always feasible due to rapid worsening of clinical conditions or the inability to postpone surgery due to the imminent need for surgical treatment.

However, our experience has taught us that with adequate organization and close multidisciplinary collaboration based on periodic checks and comparisons, many surgeries can be scheduled after the time necessary to ensure an adequate pharmacological wash-out or after an improvement of nutritional conditions.

Clearly, these attentions should be addressed to patients who do not have absolute criteria of surgical urgency such as peritonitis, massive bleeding or major occlusive states.

Our future commitment, also through specific research protocols, aims at an ever more precise and detailed preoperative stratification to identify more and more patients susceptible to preoperative optimization.

## Recurrence

Approximately 30% of patients with CD who undergo surgery develop endoscopic recurrence as early as one year after surgery [10].

In the 5 years after surgery, up to 50% of patients require repeat surgery for clinically significant recurrence [2].

Although smoking is a well-known risk factor for postoperative recurrence, in our series, this correlation was not statistically confirmed.

The role of microscopic disease involvement of the bowel margins of surgical resection is currently a topic of discussion regarding the anastomotic recurrence of disease [11].

A historical randomized trial by Fazio et al. [12] conducted on 131 patients undergoing surgery for CD influenced most of the surgical strategies adopted in the following years for the surgical management of CD.

In particular, Fazio and colleagues did not observe a significant difference in the incidence of clinically relevant recurrence in patients with or without resection margins involved with the disease. The conclusions suggested that extended intestinal resections beyond the macroscopically pathological border were not necessary and unjustified.

More recently, evidence contradicting this recommendation has been reported, largely supported by retrospective analyses, such as in the study conducted by A. de Buck van Overstraeten et al. [13] on 538 patients undergoing primary ileocecal resection for CD in two of the largest European centres.

In the present series, microscopic positivity of the margins of exeresis was a highly significant independent risk factor associated with increased surgical recurrence (HR 2.99, 1.36–6.54; *p* = 0.006).

Our experience allowed us to reach the same conclusions as A. de Buck van Overstraeten et al., who calculated an 8.7 times greater risk of developing postoperative surgical recurrence in the presence of microscopic positivity of the resection margins.

Our study certainly presents the limitations of any retrospective analysis, but it is a starting point for future prospective investigations.

However, the fear of the risk of short bowel syndrome that patients with CD may experience for various reasons (extensive disease, multiple localizations, frequent need for surgical reoperations) does not support extensive resections with radical intent, as the infiltration of the margins of exeresis is only one of the numerous, and to some extent still unknown, factors determining the risk of recurrence.

In addition to the positivity of the resection margins, our series also highlighted an increased risk of surgical recurrence in patients with a perianal localization of the disease. This evidence, which was probably conditioned by the exiguity of the sample studied, requires further targeted analyses to be confirmed and correctly interpreted.

## Limitations of the study

As already specified, our study is strongly conditioned by the retrospective nature of the analysis and by the long time interval considered.

The evidences that emerged can, however, serve, to us as to other researchers, as a starting point to study in deep some fundamental issues in CD surgery such as the role of risk factors for postoperative complications and the possibility, in some cases, of intervening preoperatively to improve these factors.

Further prospective studies are needed to validate and clarify the role of the emerging evidences.

## Conclusions

Crohn’s disease poses two important challenges for the surgeon: the high postoperative complication rate and the disease recurrence that requires new surgical treatment.

Long-term treatments with steroid drugs, administered up to 7 days before surgery, increases the overall risk of postoperative complications by approximately 3 times. Preoperative clinical malnutrition, which is frequent in these patients due to malabsorption, short bowel syndrome or recurrent subocclusive episodes, compromises the postoperative course.

Our data revealed how therapeutic choices and preoperative measures (e.g. suspension of steroid therapy and preoperative nutritional optimization) might significantly improve early and late outcomes. Unfortunately, the severity of the clinical picture may occasionally force the surgeon to work with a very limited window for preoperative optimization.

Microscopic inflammatory involvement of the margins was the only surgical variable able to increase the risk of surgical recurrence in our patients, but further prospective studies are necessary to definitively clarify its importance.

Our study has confirmed the importance of treating patients with CD in high-volume reference centres where experienced physicians pay attention to the history and clinical conditions of each patient to correctly define the proper surgical timing.

A “tailored” treatment is necessary to optimize the preoperative status, improve short-term outcomes, and reduce the risk of clinical recurrence.

## Data Availability

The datasets generated during and/or analyzed during the current study are available from the corresponding author on reasonable request.
